# The Fate of miRNA* Strand through Evolutionary Analysis: Implication for Degradation As Merely Carrier Strand or Potential Regulatory Molecule?

**DOI:** 10.1371/journal.pone.0011387

**Published:** 2010-06-30

**Authors:** Li Guo, Zuhong Lu

**Affiliations:** 1 State Key Laboratory of Bioelectronics, School of Biological Science and Medical Engineering, Southeast University, Nanjing, China; 2 Key Laboratory of Child Development and Learning Science of Ministry of Education, Southeast University, Nanjing, China; University of California Riverside, United States of America

## Abstract

**Background:**

During typical microRNA (miRNA) biogenesis, one strand of a ∼22 nt RNA duplex is preferentially selected for entry into a silencing complex, whereas the other strand, known as the passenger strand or miRNA* strand, is degraded. Recently, some miRNA* sequences were reported as guide miRNAs with abundant expression. Here, we intended to discover evolutionary implication of the fate of miRNA* strand by analyzing miRNA/miRNA* sequences across vertebrates.

**Principal Findings:**

Mature miRNAs based on gene families were well conserved especially for their seed sequences across vertebrates, while their passenger strands always showed various divergence patterns. The divergence mainly resulted from divergence of different animal species, homologous miRNA genes and multicopy miRNA hairpin precursors. Some miRNA* sequences were phylogenetically conserved in seed and anchor sequences similar to mature miRNAs, while others revealed high levels of nucleotide divergence despite some of their partners were highly conserved. Most of those miRNA precursors that could generate abundant miRNAs from both strands always were well conserved in sequences of miR-#-5p and miR-#-3p, especially for their seed sequences.

**Conclusions:**

The final fate of miRNA* strand, either degraded as merely carrier strand or expressed abundantly as potential functional guide miRNA, may be destined across evolution. Well-conserved miRNA* strands, particularly conservation in seed sequences, maybe afford potential opportunities for contributing to regulation network. The study will broaden our understanding of potential functional miRNA* species.

## Introduction

**Figure 1:**
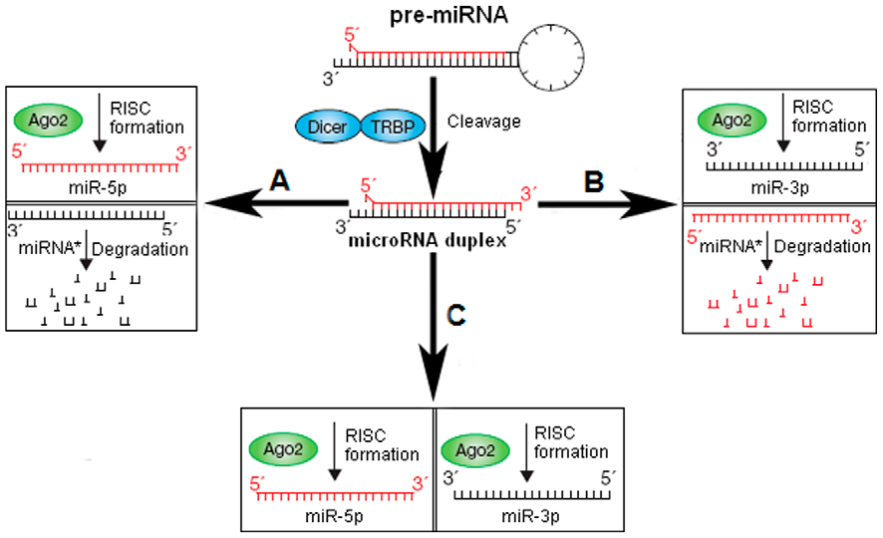
The selection of mature miRNA strand and the fate of its passenger strand. (A) The mature miRNA sequence is miR-#-5p, and its passenger sequence is miRNA* (star) which is degraded. (B) The mature miRNA sequence is miR-#-3p, and its passenger sequence is miRNA*. (C) Both the miR-#-5p and miR-#-3p are mature miRNA sequences that guide RISC to silence target mRNAs through mRNA cleavage, translational repression or deadenylation.

MicroRNAs (miRNAs) are an abundant class of small non-protein-coding RNAs that have emerged as key post-transcriptional regulators of gene expression in animals and plants [1], [2]. Metazoan miRNA genes are transcribed by either RNA polymerase II or RNA polymerase III into primary miRNA transcripts (pri-miRNAs) as single genes or in clusters [1], [3], [4], [5]. The pri-miRNAs contain stem-loop structures (hairpins) that harbor the miRNAs in the 5' or 3' half of the stem. These primary miRNA gene transcripts are typically, but not always, recognized and cut by the endonuclease Drosha in the cell nucleus to produce miRNA hairpin precursors that are then exported to the cytosol, where the hairpin structures are cut by the endonuclease Dicer at relatively fixed positions and released as short double-stranded RNA duplexes [6], [7], [8], [9], [10], [11], [12]. Although both strands of duplexes are necessarily produced in equal amounts by transcription, their accumulation is asymmetric at steady state [13]. Based on the thermodynamic stability of each end of this duplex, one of the strands is thought to be a biologically active miRNA, and the other is considered as an inactive strand and a carrier strand called miRNA* (miRNA star) or passenger strand [14]. Generally, the miRNA* strand is typically degraded, whereas the mature miRNA strand is taken up into the microribonucleoprotein complex (miRNP) [6] (Figure 1A and Figure 1B). The mature miRNA strand is used as a guide to direct negatively post-transcriptional regulation by the binding of 5'-seed (nucleotides 2–8) and anchor (nucleotides 13–16) with target sequences in the 3' untranslated region (UTR) of cognate mRNAs [1], [15]. Once bound to Ago proteins, miRNAs are more stable than average mRNAs and the half-life of most miRNAs is greater than 14 hours [16]. They may be produced by 5' (left arms) or 3' arms (right arms) of the miRNA precursors, and the nonrandom nature of miRNA strand selection might reflect an active process that minimizes the population of silencing complexes with illegitimate miRNA* species [13] (Figure 1). The mechanism of strand selection maybe correlates with the relative free energies of the duplex ends [11], [13], [17].

However, recently, some miRNA* sequences were reported as mature functional miRNAs with abundant expression, and miRNA/miRNA* ratios may vary dramatically among developmental stages [13], [18], [19]. Most *Drosophila* miRNAs are bound to Ago1, and miRNA* strands accumulate bound to Ago2 [20]. The rarer partner of the mature miRNA, has been recognized both in terms of increasing the complexity of regulatory networks and in governing miRNA and messenger RNA evolution [13], [19], [21], [22], [23]. Some hairpins produce miRNAs from both strands at comparable frequencies because strand selection is often not a stringent process [24]. These abundant miRNA* species are often present at physiologically relevant levels and can associate with Argonaute proteins [13] (Figure 1). During Drosophilid evolution, more than 40% miRNA* sequences resist nucleotide divergence, and at least half of these well-conserved miRNA* species select for conserved 3' untranslated region seed matches well above background noise [13]. The miRNA* species diverge much more slowly than miRNA terminal loops, and conserved miRNA* sequences are almost perfectly conserved in sequences similar to mature miRNA sequences [13], [22]. According to miRBase database (version 14.0, http://www.mirbase.org/), about 80 kinds of human miRNA precursors can yield two kinds of abundant mature miRNAs (left-arm, miR-#-5p; right-arm, miR-#-3p) with different seed sequences and target mRNAs, while most miRNA precursors only yield abundant mature miRNAs from left-arms or right-arms and rare miRNA* sequences. Most miRNA* species still keep fewer sequence counts despite of their mature miRNA sequences are detected higher expression levels based on high-throughput method [25]. Therefore, those specific miRNA precursors that yield two kinds of abundant functional miRNAs from different arms maybe reflect evolutionary implication across miRNA gene evolution. Although evolutionary patterns of miRNA* are consistent with their regulatory potential across Drosophilid evolution [13], limited knowledge about evolutionary information of miRNA/miRNA* has been discussed especially across different animal species.

miRNAs are evolutionary conserved across broad phylogenetic distances [26], [27], [28], and they have gained considerable attention about evolution, genetic and phylogenetic analysis [15], [23], [29], [30], [31], [32], [33]. The non-coding small RNAs are strongly conserved in primary sequence and rarely secondarily lost once integrated into a gene regulatory network [22], [31], [34]. Recent study suggested an explosive increase in the miRNA repertoire in vertebrates [35]. Some miRNAs in a single animal species are similar in sequence and produce the same or similar mature miRNA sequences, and these miRNAs always compose miRNA gene family. These family members may be derived from ancestral miRNA gene directly or indirectly through duplication, but the duplication process maybe complex and unclear based on limited miRNA data across animal species. Nonetheless, miRNA gene evolution might provide potential implication for selection of miRNA and fate of miRNA*. The miRNA* strand with lower expression level because of degradation, or as functional mature miRNA with abundant clones, maybe get evolutionary implication by analyzing their evolutionary patterns. In the study, we intended to discover potential relationship between evolutionary pattern and selection of mature miRNA by analyzing miRNA/miRNA* based on miRNA gene families and single miRNA gene across vertebrates. Simultaneously, we also analyzed a complex miRNA gene family from a single animal species to study divergence trends of miRNA/miRNA* and discover potential evolutionary implication across evolution. Finally, because different miRNAs showed different distribution spectrums and evolutionary patterns across vertebrates, evolutionary analysis of miRNA/miRNA* based on single miRNA gene was performed across the same kinds of typical animals.

## Results

### Divergence patterns of miRNA/miRNA* based on miRNA gene families

**Figure 2:**
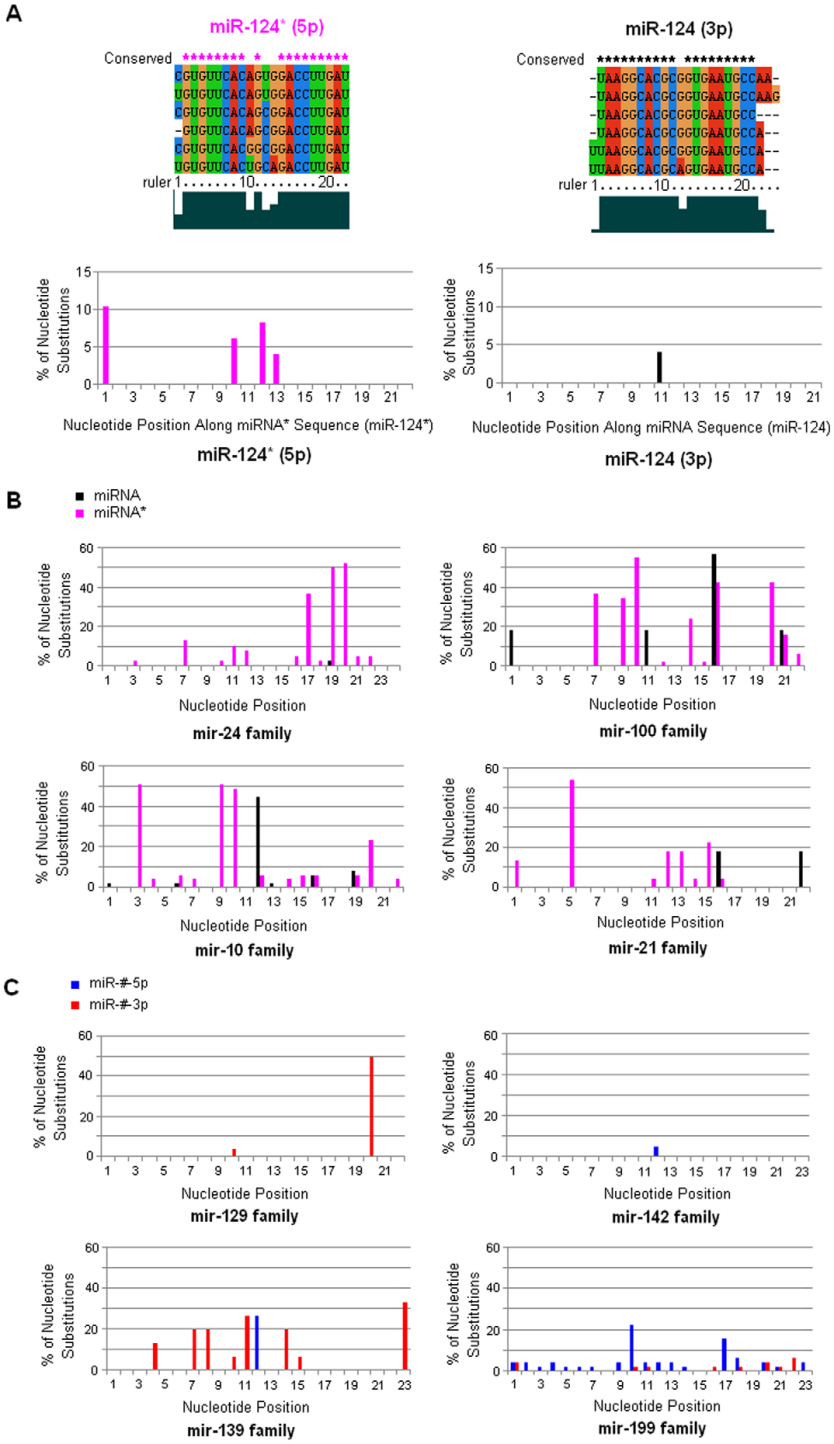
Clustal multiple sequence alignment and mutational profile of miRNA and miRNA* sequences across vertebrates. (A) Multiple sequence alignment of mir-124 family and mutational profile of miRNA and miRNA* sequences. Repeated sequences were discarded. Conserved: conserved positions in sequence alignment. The bottom figures were the percentage of nucleotide substitutions from positions 1 to ∼22 in miRNA and miRNA* sequences. Both miR-124 and miR-124* sequences were well conserved, especially miR-124. (B) Mutational profile of miRNA/miRNA* in miRNA gene families. Both miRNA and miRNA* sequences showed different evolutionary patterns. miRNA* sequences always were involved more nucleotide substitutions than miRNAs, even in positions 2–8. (C) Mutational profile of miR-#-5p/miR-#-3p in miRNA gene families. These miRNAs (miR-#-5p and miR-#-3p) were reported as abundant miRNAs according to miRBase database (version 14.0). Although miR-#-5p had different divergence pattern with miR-#-3p, both of them always were well conserved especially for the seed sequences. In mir-199 family, nucleotide divergences of miR-199-5p mainly resulted from gga-mir-199b and bta-mir-199c.

Mature miRNAs always were highly conserved across vertebrates, especially in seed sequences (nucleotides 2–8) and anchor sequences (nucleotides 13–16), while their passenger strands showed higher nucleotide divergence (Figure 2). Some miRNA* species were well conserved across vertebrates although they showed a higher level of nucleotide divergence than their partners (Figure 2A and Figure 2B). The divergence mainly resulted from divergence of different animal species, homologous miRNA genes and multicopy hairpin precursors. For example, in complex miRNA gene families, such as let-7 family, miRNA* sequences were less conserved because of wide distribution spectrum in vertebrates, multiple homologous genes and multicopy precursors. Different miRNA* sequences showed different levels of nucleotide divergence. miR-124* sequences were well conserved, while miR-100* and miR-10* sequences showed greater nucleotide divergences than their miRNAs (Figure 2A and Figure 2B). Even in positions 2–8, some miRNA* sequences were involved nucleotide substitutions. According to miRBase database, some miRNA precursors were reported that they could generate two kinds of abundant miRNAs (miR-#-5p and miR-#-3p). Intriguingly, despite involved homologous genes and multicopy precursors (such as mir-142 family and mir-129 family), many of these miR-#-5p/miR-#-3p sequences were well conserved (Figure 2C). However, miR-#-5p and miR-#-3p showed different levels of nucleotide divergence despite both of them always had conserved seed sequences.

**Figure 3:**
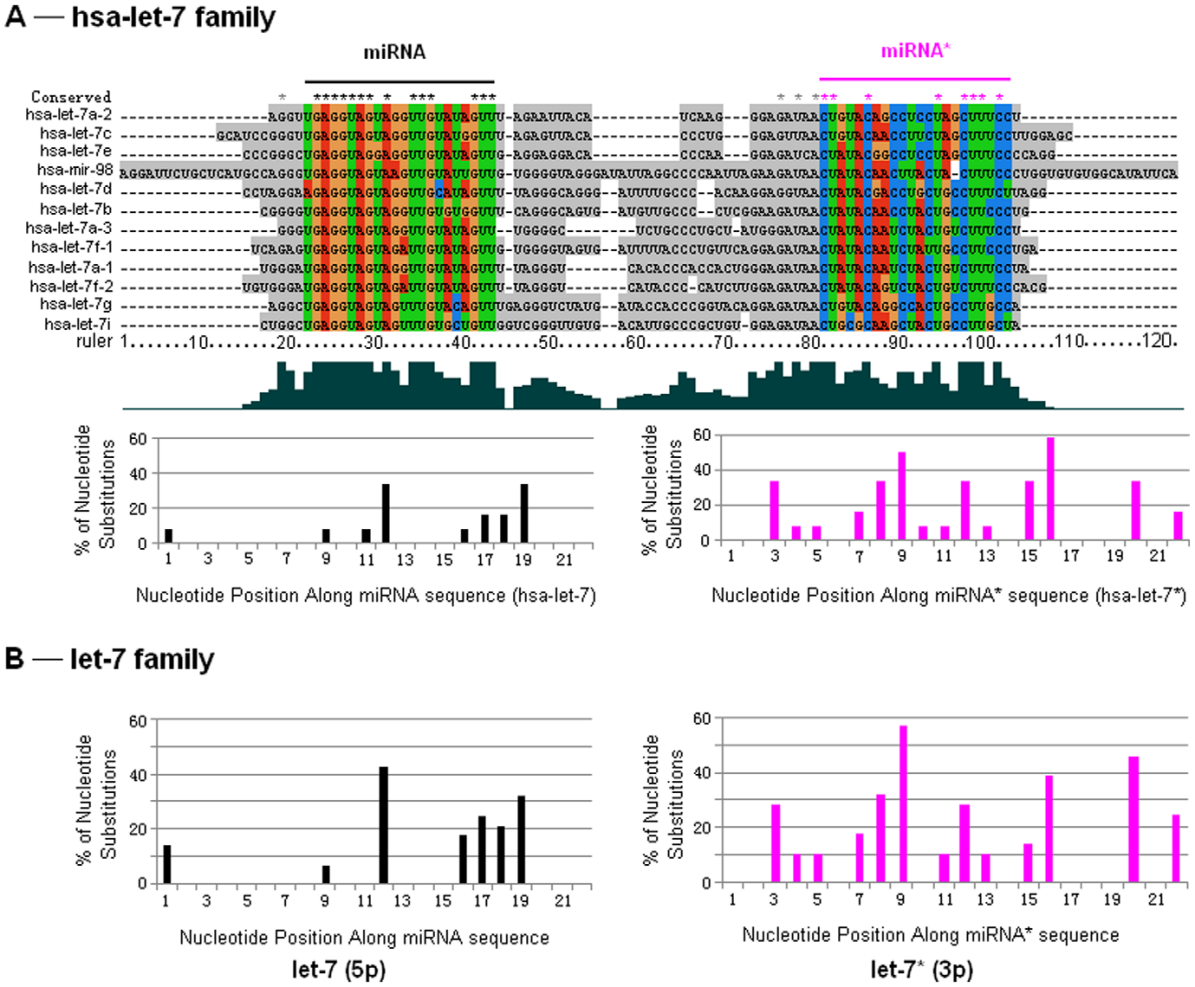
Clustal multiple sequence alignment of let-7 family and mutational profile of miRNA and miRNA* sequences. (A) Hsa-let-7 showed high conservation in positions 2-8 (the seed sequence) and relatively conservation in positions 13–16, while miRNA* sequence showed frequent substitutions and insertions/deletions. (B) Let-7 sequences across vertebrates showed high conservation in positions 2–8 (the seed sequence), while let-7* sequences showed frequent substitutions.

**Figure 4:**
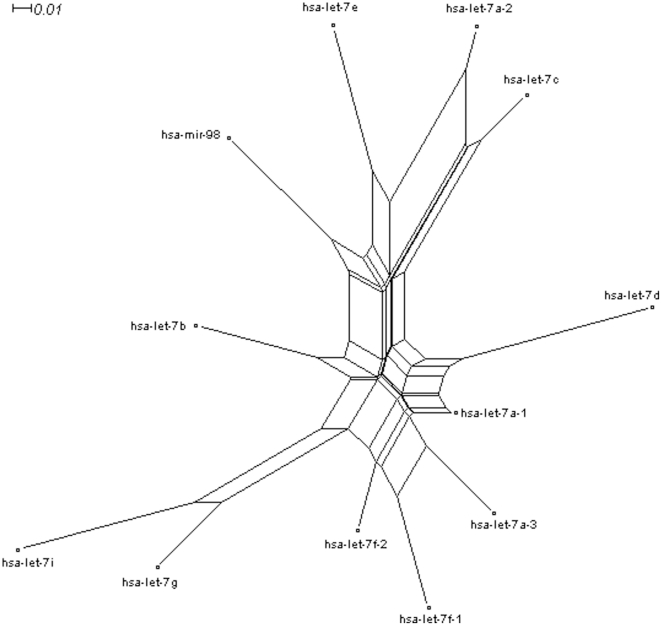
Phylogenetic network of hsa-let-7 family using neighbor-net method. Based on miRNA precursor sequences of let-7 family in *Homo sapiens*, 12 miRNA genes were split into several clusters. Multicopy miRNA precursors for a single miRNA, for example, hsa-let-7a-1, hsa-let-7a-2 and hsa-let-7a-3, were reconstructed in different clusters.

Although miRNA gene families may be involved complex evolutionary history across the animal kingdom and in a single animal species, miRNA/miRNA* based on a single animal species might show different levels of nucleotide divergence and imply different fates. Here, we took an example of let-7 family in *Homo sapiens*, which included several homologous members (Figure 3A). Some of these members could be found to have multicopy precursors, for example, hsa-let-7a could be produced by hsa-let-7a-1, hsa-let-7a-2 and hsa-let-7a-3. Mature hsa-let-7 sequences were produced by 5p (left arms) and well conserved especially for the seed sequences (nucleotides 2–8) and anchor sequences (nucleotides 13–16), while hsa-let-7* showed a higher level of nucleotide divergence even positions 2–8 (Figure 3A). These multicopy precursors could yield the same mature miRNA sequences, but their loop sequences and miRNA* strands might show greater divergence than miRNAs (Figure 3A). Interestingly, the similar trend of nucleotide divergence of miRNA and miRNA* could be detected across vertebrates (Figure 3B). Phylogenetic network of hsa-let-7 family was split into several clades based on different miRNA genes (Figure 4). Multicopy precursors for a single miRNA, such as hsa-let-7a-1, hsa-let-7a-2 and hsa-let-7a-3, might be reconstructed in different clusters (Figure 4).

### Divergence patterns of miRNA/miRNA* based on single miRNA gene

We observed different amounts of nucleotide divergence between miRNA and miRNA* sequences based on single miRNA gene, such as miR-125a-5p/miR-125a-3p and miR-210/miR-210* (Figure S1). Generally, more sites of miRNA* were involved divergence despite miRNAs were highly conserved (Figure S1). Different levels of divergence pattern also were detected in mammalian-specific miRNAs. Similarly, loop sequences showed different levels of divergence between various miRNA genes (Figure S1). According to human miRNAs in miRBase database, 80 kinds of miRNA precursors were reported two kinds of abundant miRNAs (miR-#-5p and miR-#-3p). Sequence analysis based on miRNA precursor sequences revealed that >80% of these miR-#-5p and miR-#-3p sequences ensured conserved seed sequences throughout evolution.

**Figure 5:**
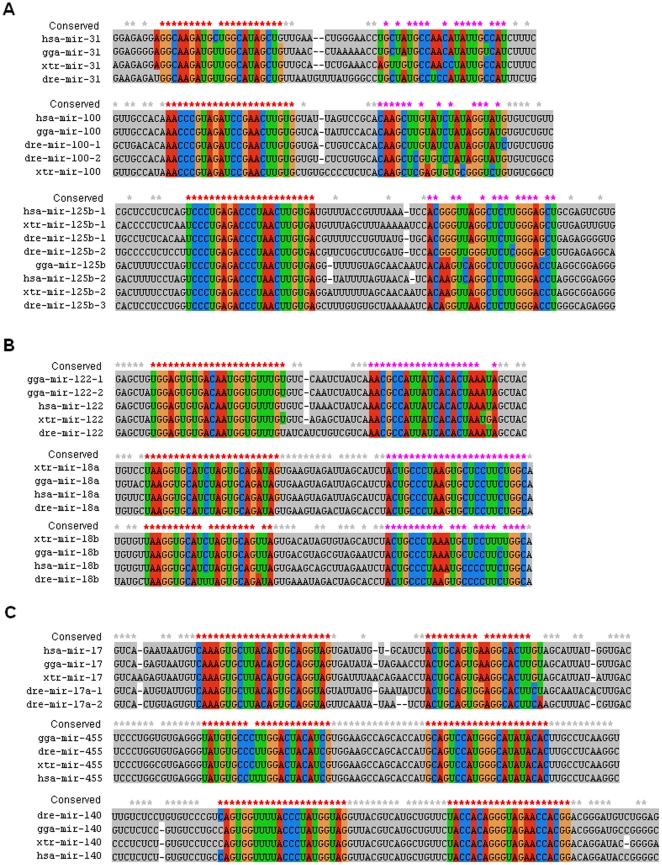
Nucleotide divergences of miRNA and miRNA* across several typical vertebrates. In order to avoid influence of various distribution spectrums of miRNAs, we selected several typical vertebrates to analyze miRNA evolutionary trends: dre (*Danio rerio*, Pisces), hsa (*Homo sapiens*, Mammalia), gga (*Gallus gallus*, Aves) and xtr (*Xenopus tropicalis*, Amphibia). Red conserved sites indicated mature miRNAs (including less abundant but functional miRNA* strands), pink conserved sites indicated miRNA* sequences that were not reported as functional guide miRNAs. Some flank sequences of miRNA sequences were deleted. All the mature miRNAs were highly conserved in these animals, while their miRNA* strands showed different divergence trends. (A) miRNA* sequences were less conserved due to divergence of different animals and multicopy miRNA precursors. (B) miRNA* sequences were well conserved similar to mature miRNAs though they were not reported as abundant functional miRNAs. (C) miR-#-5p and miR-#-3p were well conserved across different animals, and both of them were reported as abundant functional guide miRNAs.

Because miRNAs always showed different distribution spectrums across the animal kingdom, we also analyzed miRNA and miRNA* across several kinds of typical vertebrate animals: *Danio rerio* (Pisces), *Homo sapiens* (Mammalia), *Gallus gallus* (Aves) and *Xenopus tropicalis* (Amphibia). Mature miRNAs were highly conserved across these animal species, while their passenger strands showed different evolutionary patterns (Figure 5). Some miRNA* sequences were less conserved even in positions 2–8, such as miR-31*, miR-100* and miR-125b*, while their terminus regions (5' and 3') were more conserved than their central regions. Other miRNA* sequences were well conserved similar to mature miRNAs, such as miR-18a*, miR-18b*, miR-17-3p and miR-455-3p (Figure 5). Although mature miR-100 and miR-125b were highly conserved, their star sequences showed greater divergence across species. Some well-conserved miRNA* species were reported as functional guide miRNAs with abundant expression, which were well conserved particularly in seed and anchor sequences (Figure 5C). Homologous miRNA genes maybe showed different divergence patterns in the same kinds of animals, such as miR-18a* and miR-18b* (Figure 5B). The loop sequences also showed different divergence trends although they maybe showed greater divergence than miRNA and miRNA*(Figure 5).

## Discussion

Mature miRNAs (miR-#-5p or miR-#-3p) were evolutionarily conserved across the animal kingdom [26], [27], [28], while their passenger strands, either as typically degraded miRNA* or abundant mature miRNAs, always showed conservation across vertebrates with higher nucleotide divergences than their partners (Figure 2). Different miRNA* sequences showed various divergence patterns. Data analysis revealed that some mature miRNAs and their passenger strands were well-conserved, especially in their seed sequences (Figure 2). For example, miR-124 (Figure 2A), a phylogenetic conserved miRNA from *Caenorhabditis* to *Homo*, is one of the most abundantly expressed miRNAs in the nervous system and contributes to the development of nervous system [36], [37], [38]. However, some miRNA* sequences showed higher level of nucleotide divergence, even though in their positions 2–8, such as miR-10* and miR-100* (Figure 2B). Even if multicopy hairpin precursors could yield the same mature miRNA sequences, another product, termed as miRNA* species, always diverged, such as hsa-let-7a-1* and hsa-let-7a-2*, hsa-let-7f-1* and hsa-let-7f-2* (Figure 3A). Despite of greater divergence than miRNAs, we also found miRNA* diverged much more slowly than terminal loops, which maybe strongly aid the identification of functional animal miRNA hairpins as “saddle” structures [39], [40]. Interestingly, similar divergence trends of human let-7/let-7* could be detected by sequence analysis across vertebrates (Figure 3), which might reveal historical miRNA gene divergence and similar evolutionary trend across different animals. High divergence levels could be detected among these homologous miRNA genes (Figure 3 and Figure 4). Although the divergence mainly resulted from the loop regions, the divergence of miRNA* strands also contributed partly to the high divergence level (Figure 3A and Figure 4). On the other hand, we selected several typical vertebrate animals to analyze miRNA/miRNA* sequences because different miRNAs had different distribution spectrums across the animal kingdom. Similarly, some miRNA* strands were highly conserved, but others were less conserved despite their mature miRNAs were well conserved (Figure 5). Those miRNAs that reported both miR-#-5p and miR-#-3p could be mature functional miRNAs always were well conserved especially for seed and anchor sequences, such as miR-17, miR-140 and miR-455 (Figure 5). Nevertheless, some miRNA* strands were diverged even in their seed sequences, such as miR-31*, miR-100* and miR-125b* (Figure 5). Therefore, across miRNA gene evolution, functional mature miRNAs still were well conserved especially for their seed sequences, while miRNA* sequences showed various evolutionary patterns. Some miRNA* maybe showed high divergence levels between different precursors even between different multicopy precursors, but others ensured well-conserved seed sequences, especially for those miRNA genes that generated abundant miRNAs from two arms of hairpins (Figure 2, Figure 5 and Figure S1). Evolutionary conservation of passenger strand might result from two plausible reasons. Firstly, evolutionary process would be influenced because it maybe contributed to stable stem-loop structure of miRNA hairpin precursor. Secondly, the well conservation of passenger strand might afford an opportunity to be mature miRNA to bind target mRNA similar to its partner. Therefore, the evolutionary patterns of miRNA* might be a pivotal implication (discussed below).

According to miRNA biogenesis, as miRNA partners, the miRNA passenger strands should be more tightly constrained at their 3' ends which pair with the miRNA seed sequences (nucleotides 2–8). However, similar to Okamura et al. [13], systematic analysis showed that some miRNA* sequences were notably analogous to miRNA strands: well conserved in seed (nucleotides 2–8) and anchor sequences (nucleotides 13–16) (Figure 2, Figure 5 and Figure S1). They also showed patterns of nucleotide divergence that were consistent with their selection for regulatory activity [13]. Therefore, the evolutionary pattern of miRNA* afforded an opportunity to be abundant functional guide miRNAs based on well conserved seed sequences that reflected their sequence-based, trans-regulatory activity [13], [41]. Indeed, earlier computational efforts for miRNA genes finding hinted the possibility of trans-acting activity for miRNA* species [40]. Some miRNA* strands were abundant because they were degraded more slowly than others, and the miRNA:miRNA* ratio of many loci became increasingly skewed as development proceeded [13]. According to miRBase database, we found two kinds of mature products (miR-#-5p from left-arm and miR-#-3p from right-arm) were reported from some miRNA precursors, such as mir-199a and mir-17. Analysis of miRNA based on high-throughput sequencing data also showed abundant miRNA* although less abundant than their partners [25]. Recent study revealed that miRNA:miRNA* ratios were flexible in different development stages and both of them resisted nucleotide divergence across Drosophilid evolution [13]. The expression level of miRNA passenger strand mainly relied on degradation degree and degradation rate, because both strands of miRNA duplex were necessarily produced in equal amounts by transcription. We found different miRNA* showed various divergence patterns despite their mature miRNAs were highly conserved (Figure 2, Figure 5 and Figure S1). Generally, those less conserved miRNA* strands were not reported as mature functional miRNAs. The divergence of less-conserved miRNA* always resulted from individual animal and/or multicopy precursors (Figure 5 and Figure S1). Evolutionary trends of the miRNA* strands might be potential implication for their final fates: degradation as by-products or functional regulatory molecules as mature miRNAs. It is plausible that non-functional miRNA* strands maybe involved higher rates of nucleotide substitution during evolution, while functional miRNA* sequences would be strictly regulated that were critical during binding target mRNAs. The correlation between the evolutionary constraint of miRNA* and their expression levels might reflect their potential function as endogenous regulatory RNAs. Some miRNA* strands might become functional guide strands and they were phylogenetically conserved similar to their mature miRNAs. Those well conserved miRNA* strands might also play important roles in regulating network in different development stages, but limited miRNA data cannot afford enough experimental evidences. Therefore, evolutionary patterns of many miRNA* strands were consistent with their regulatory potential [13], [23], and the final fate, degradation as merely carrier strand or becoming potential functional guide miRNAs, might be got some implication throughout miRNA gene evolution. Some passenger strands were well-conserved in positions 2–8 similar to their mature miRNAs, and the phylogenetic conservation of miRNA* may be evolutionary implication to become abundant guide miRNAs and play important roles in particular developmental contexts at specific times. The systematic evolutionary analysis maybe broaden our understanding of miRNA* strands, especially for those potential regulatory miRNA* species.

## Materials and Methods

All the miRNA and miRNA* sequences, and their miRNA precursor sequences from different animal species were obtained in miRBase database (version 14.0, http://www.mirbase.org/). We denoted the miRNA precursors by mir-#, the mature miRNAs by miR-#, and miRNA* (miRNA star) by miR-#* in accordance with the convention in miRBase database. If the miRNA* strands were reported as abundant mature miRNA, miR-#-5p or miR-#-3p was denoted. In the study, miR-#-5p and miR-#-3p were identified according to human miRNAs in miRBase database. These sequences were aligned with Clustal X 2.0 [42] by using the multiple sequence alignment. Phylogenetic network of miRNA genes was reconstructed using the neighbor-net method [43] based on Jukes-Cantor model as implemented in SplitsTree 4.10 [44]. For human let-7 family, we attempted to reconstruct the evolutionary history from the gene tree and discover potential evolutionary implications of let-7 and let-7*. All the gaps/missing data were deleted in phylogenetic network.

Because miRNA* sequences always degraded, there were limited miRNA* sequences in miRBase database. In order to discover detailed evolutionary information, we analyzed predicted consensus sequences as miRNA* sequences according to known miRNA* based on their precursor sequences. Because of imprecise and alternative cleavage of Dicer and Drosha, multiple isomiRs, the population of variants of known miRNAs, have been identified from the sequencing data by applying high-throughput DNA sequencing technologies [25], [45], [46], [47], [48]. Therefore, in the study, we only analyzed nucleotide substitutions of internal sequences of miRNA and miRNA* without considering gaps/missing sites in the terminus regions. Percentage of nucleotide substitution at positions (from 1 to ∼22) was estimated for miRNAs and miRNA* sequences by analyzing all the miRNA precursors from miRBase database. In order to estimate substitution trend more precisely, we selected the most abundant nucleotide at each position as reference nucleotide.

## Supporting Information

Figure S1 Patterns of nucleotide divergence of miRNA and miRNA* across vertebrates. (A) and (B) showed well conserved miR-#-5p and miR-#-3p based on miRNA gene family. (C) and (D) showed divergence patterns of miR-#-5p/miR-#-3p and miRNA/miRNA* based on single miRNA gene.
(3.99 MB TIF)
