# Supplementary figures and images for: The Fate of miRNA* Strand through Evolutionary Analysis: Implication for Degradation As Merely Carrier Strand or Potential Regulatory Molecule?
Source: PLoS One. 2010 Jun 30;5(6):e11387. doi: 10.1371/journal.pone.0011387 (PMC2894941; doi:10.1371/journal.pone.0011387)

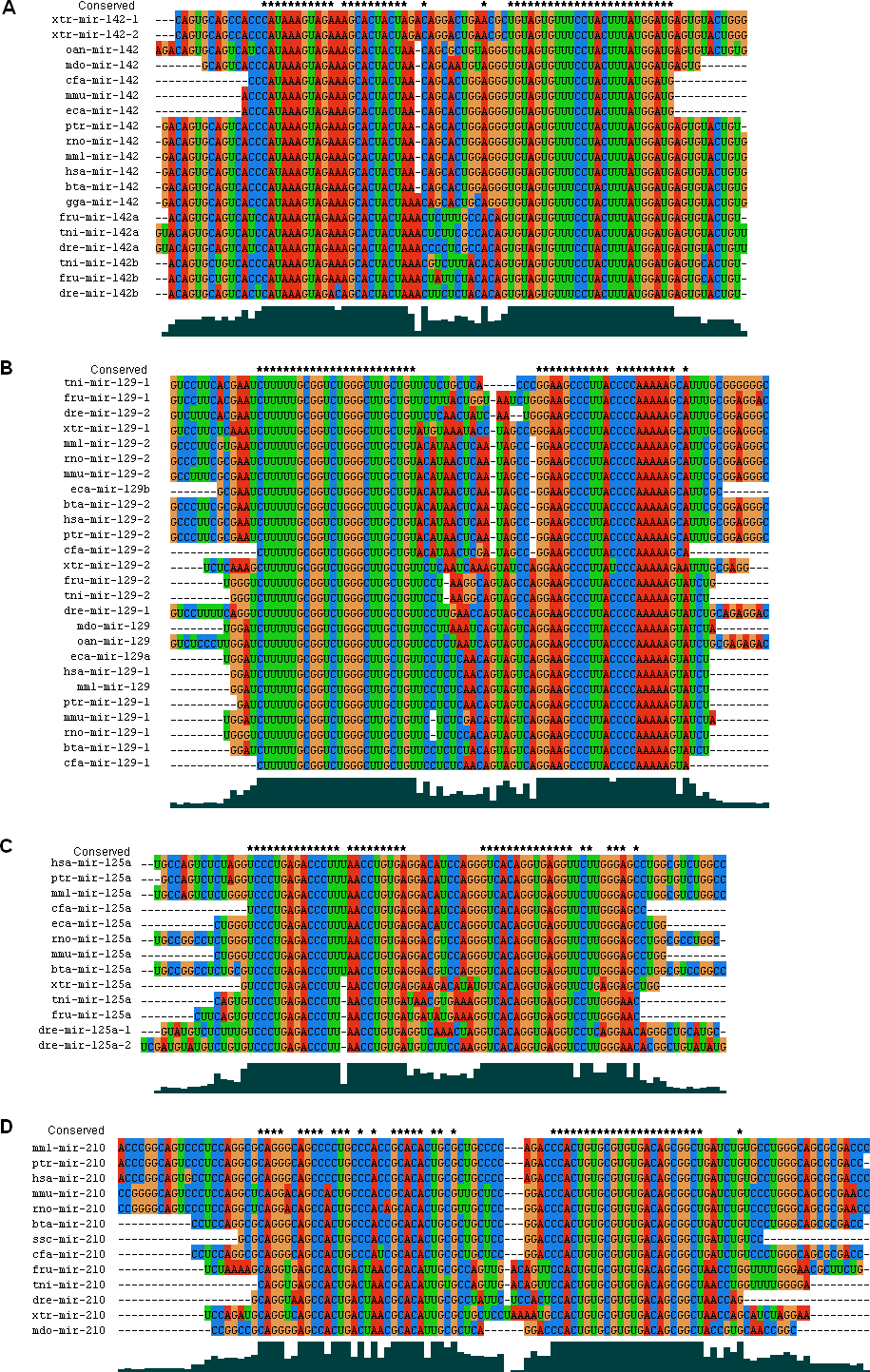

Supplement: Figure S1 — Patterns of nucleotide divergence of miRNA and miRNA* across vertebrates. (A) and (B) showed well conserved miR-#-5p and miR-#-3p based on miRNA gene family. (C) and (D) showed divergence patterns of miR-#-5p/miR-#-3p and miRNA/miRNA* based on single miRNA gene. (3.99 MB TIF) [file pone.0011387.s001.tif]
